# TANVEER: Tri-Angular Nearest Vector-Based Energy Efficient Routing for IoT-Enabled Acoustic Sensor and Actor Networks (I-ASANs)

**DOI:** 10.3390/s21113578

**Published:** 2021-05-21

**Authors:** Umar Draz, Sana Yasin, Muhammad Irfan, Tariq Ali, Amjad Ali, Adam Glowacz, Frantisek Brumercik, Witold Glowacz

**Affiliations:** 1 Computer Science Department, COMSATS University Islamabad, Lahore Campus, Lahore 54000, Pakistan; sheikhumar520@gmail.com (U.D.); amjad.ali@cuilahore.edu.pk (A.A.); 2 Department of Computer Science, University of Sahiwal, Sahiwal 57000, Pakistan; 3 Department of Computer Science, University of Okara, Okara 56300, Pakistan; sanayaseen42@yahoo.com; 4 Electrical Engineering Department, College of Engineering, Najran University, Najran 61441, Saudi Arabia; miditta@nu.edu.sa; 5 Computer Science Department, COMSATS University Islamabad, Sahiwal Campus, Sahiwal 57000, Pakistan; tariqali@cuisahiwal.edu.pk; 6 Department of Automatic Control and Robotics, Faculty of Electrical Engineering, Automatics, Computer Science and Biomedical Engineering, AGH University of Science and Technology, 30-059 Kraków, Poland; wglowacz@agh.edu.pl; 7 Department of Design and Machine Elements, Faculty of Mechanical Engineering, University of Zilina, 010 26 Zilina, Slovakia; frantisek.brumercik@fstroj.uniza.sk

**Keywords:** TANVEER, acoustic, energy consumption, empty-region, achievable region, angle-based flooding, watchman nodes, binary inter nodes

## Abstract

The Internet of Things (IoT) is an emerging technology in underwater communication because of its potential to monitor underwater activities. IoT devices enable a variety of applications such as submarine and navy defense systems, pre-disaster prevention, and gas/oil exploration in deep and shallow water. The IoT devices have limited power due to their size. Many routing protocols have been proposed in applications, as mentioned above, in different aspects, but timely action and energy make these a challenging task for marine research. Therefore, this research presents a routing technique with three sub-sections, Tri-Angular Nearest Vector-Based Energy Efficient Routing (TANVEER): Layer-Based Adjustment (LBA-TANVEER), Data Packet Delivery (DPD-TANVEER), and Binary Inter Nodes (BIN-TANVEER). In TANVEER, the path is selected between the source node and sonobuoys by computing the angle three times with horizontal, vertical, and diagonal directions by using the nearest vector-based approach to avoid the empty nodes/region. In order to deploy the nodes, the LBA-TANVEER is used. Furthermore, for successful data delivery, the DPD-TANVEER is responsible for bypassing any empty nodes/region occurrence. BIN-TANVEER works with new watchman nodes that play an essential role in the path/data shifting mechanism. Moreover, achievable empty regions are also calculated by linear programming to minimize energy consumption and throughput maximization. Different evaluation parameters perform extensive simulation, and the coverage area of the proposed scheme is also presented. The simulated results show that the proposed technique outperforms the compared baseline scheme layer-by-layer angle-based flooding (L2-ABF) in terms of energy, throughput, Packet Delivery Ratio (PDR) and a fraction of empty regions.

## 1. Introduction

The Earth’s surface, around 71.2% is covered by ocean. Approximately half of the population of this world lives under 100 km of seaside areas. Furthermore, the ocean has been a source of nutrition products, and over time, has begun playing a vital role in the areas of defense, carriage, natural resources, and leisure. While water is indispensable for all humankind, a surprisingly massive area of the total volume of the ocean remains unexplored, i.e., less than 10% of it has been studied [1]. It is difficult for human beings to monitor volatile underwater events and high-water pressure. Because of the reasons mentioned earlier, unmanned exploration is necessary. As a result, Internet of Things-enabled acoustic sensor and actor networks (I-ASANs) have attracted the interest of researchers of late.

In the last 10 years, much research has been conducted underwater for multiple purposes [2]. Due to potential underwater applications, this research is not limited to the exploration of finding minerals, oils, and monitoring of aquatic life but also is an experimental laboratory for acoustic experiments. Today, many real-time problems have been experimented on under water such that to predict floods and tsunami, scientific and commercial offshore exploration is conducted in deep and shallow water, etc.

Many routing protocols work successfully in terrestrial wireless sensor networks (TWSNs) that cannot work steadily in underwater domains due to different communication channels like radio signals, which do not propagate underwater as this is only possible for acoustic signals. The topology of ASANs [3] is usually dynamic compared to TWSNs due to the deployment of frequent nodes and their localization. As these nodes move with mobile water [4], so energy is limited, and it is difficult to recharge the power resources in deep water. Therefore, high propagation delay and deployment cost, low bandwidth, and packet loss ratio are significant challenges in ASANs [5].

All the above challenges can be resolved if the data is smooth and sent rapidly towards the destination. Many well-known routing protocols are worked in this regard, like vector-based flooding [5] and angle-based flooding [6,7] which calculate the angle with multi-sink architecture for reliable data delivery, but the efficient way does not achieve due to week support of intermediate nodes. The unavailability of the next intermediate nodes mean empty regions are created in the network, as well as data loss of previous nodes. In an upward direction, every sensor node computes the angle that is π/2. On the basis of the angle, as mentioned earlier, the nodes forward the data packets. Data packets that reach any one of the surface sinks will be considered successfully delivered to the destination. The empty region is defined as the area in which nodes (usually present around the sink called sink neighboring nodes) fail to send the information towards the destination and create empty regions in this area. For these regions, the network traffic is stopped for the time being and isolate some part of the network. Ultimately data packet delivery and throughput is decreased and an extra energy tax is used for rescue the network especially in a large-scale area network like under water. Many secondary and rescue nodes are introduced inside the sink to recover the data [7,8].

In [8], the watchman nodes are introduced to the recovery of lost data from the empty region of the underwater network in which the watchman was continuously monitoring the node’s location and its energy status. However, these two approaches follow the monitoring approach, where the status of every node is checked by watchman nodes and corresponding angles of forwarding. The related parameters of the watchman are at distance from the node and angle cone for angle-based flooding. Both these parameters have a significant reason for high propagation and end-to-end delay, respectively. Thus, to avoid the empty region, multiple parameters need to be considered for forwarding the data packets in a well-disciplined manner. Hence, energy-efficient routing schemes are required, which have the ability to take robust short and quick routing paths between source and master stations and also avoid empty regions.

To make the scheme energy efficient, geographic routing [9] is best suited as it is frequently used due to its scalability and simplicity; also, it does not need and complete route information between two junctions. Angle-based routing is easily coped with transmitting data by circling more multiple neighbors’ nodes by computing the angles. For this, priorities are defined for different parameters like the shortest distance and energy. The nodes with the highest priority receive the data and are able to send it further. The rest of the node does not receive data because acknowledgment is received with the highest priority nodes.

Furthermore, geographical routing has a major reason to create the empty region because one node at a time is selected for transmission, and angle-based routing selects those nodes that cover with angle cone, but it consumes a lot of time and energy to compute the angle with every flooding spell. In this way, both routing schemes need to work together to cope with multiple parameters to improve energy and avoidance of empty regions.

Our Contributions. Motivated by the above consideration of energy-efficient and angle-based scenarios [6,7], we proposed the Tri-Angular Nearest Vector-Based Energy Efficient Routing (TANVEER) with its three sub-sections having exclusive features like layer-based adjustment, data-packet delivery, and binary internodes. The TANVEER, by nature, uses both angle and energy-efficient approaches for reliable communication. The layer-based adjustment of TANVEER, (LBA-TANVEER) first assigns the IDs of the nodes to all deployed nodes in the form of layers from anchored to surface of the water so that each node is easily identified with its status to detection of empty node or group of empty nodes called empty regions. All the adjustments of layers will be static throughout the network’s lifetime. Secondly, the immediate data packet delivery (DPD-TANVEER) starts whenever the empty regions are detected; the recovery process of data is done by binary inter nodes (BIN-TANVEER). The BIN is presented with the novel nodes called watchman nodes that enable data recovery from the empty regions based on the nearest vector angle is made. The contribution of our proposed scheme is given as follows: The TANVEER summarizes three metrics (three sides of angle calculation, energy, and the number of nodes) to select the nearest vector with a triangular approach to forwarding the data packets towards the sink. It selects the node which is closest to the nearest vector by flooding the tri-angular zone and finds out the closest path for the destination. The data are forwarded in the form of layer by layer manner so that each layer maintains the traffic smoothly before and after the communication. The LBA-TANVEER uses the multi-sink architecture at the surface of the water, while the topmost layer consists of ordinary sensor nodes. The watchman node with both left-right directions to rescue the empty regions are present in each layer. The DPD-TANVEER uses the same path provided by the LBA-TANVEER. The BIN-TANVEER adjusts the watchman nodes and follows the empty region’s occurrence and transfers the data through BIN towards sink through bypass the empty regions successfully. The BIN is only active when it needs, while the rest of the time, it just monitors the occurrence of empty nodes.

The rest of the paper is organized as the related latest underwater routing schemes are described in Section 2. The problem is explained in Section 3, while the proposed model and TANVEER are described in Section 4 and Section 5, respectively. Section 6, Section 7 and Section 8 deal with the proposed LBA-TANVEER, DPD-TANVEER, and BIN-TANVEER. The achievable regions are shown in Section 9 while the simulation and discussion of results are in Section 10. Trade-offs of the performance of the scheme are compared in Section 11, and Section 12 leads the conclusion.

## 2. Related Work

In this section, we summarized the different latest routing protocols in the field of ASANs with its possible pros and cons. The detailed summary of these routing protocols is given in Table 1, where each routing protocol has its achievements and limitations.

Most of the routing schemes use the direction-based flooding in which a path is established between source and destination, but the link quality does not consider directional flooding-based routing (DFR) [10]. Packets are sent according to the link quality by bypass the empty nodes while flooding. In those nodes that have a smooth path between source and master station, the packets are sent, otherwise flooding is again propagated. The main feature of the DFR is allowed at least one node participation during the detection of empty regions inside the network. It is only possible when links of the route are error-prone in terms of packet loss.

For long-term underwater environments, efficient data delivery still challenges when the nodes are sparse to each other and unpaired acoustic channels for transmission. For this, the anycast network architecture has been discussed. Two types of topology controls have been introduced in [11,12], as centralized and distributed (CTC) and (DTC). Both topologies are work with a depth control mechanism in which the data packet delivery ratio is achieved up to 90%. It is interesting to see that this technique is experimented with for both types of underwater environment, by mean very sparse and dense networks. Centralized and distributed depth adjustment with DA + CTC and DA + DTC are simulated with sonobuoys as taking as sink and provide robust communication without any occurrence of empty regions due to hybrid (centralized and distributed) approach and overall improved network lifetime.

In [9], geographic and opportunistic routing work with depth adjustment is used for communication recovery (GEDAR) when the empty region comes into existence. These strategies are mostly used with the location-based application, where oil and minerals need to be explored. Basically, provide the exact location of the occurrence of the empty region by following the greedy-based approach. As the name implies for the opportunistic routing, it always finds the early realization of the area of the node where the gaps are near. It transfers the data packets by next forwarder selection of the set for GEDAR. The limitation of location-oriented problems of the destination nodes fix the depth adjustment for each hop. As the empty regions are located, it calculates the new depth for the sonobuoys for the delivery of data. The use of multi-purpose sonobuoys in each expect neighboring node is calculated with the next hope depth so that the chances of empty regions are minimum as expected. By calculating the depth at each node, the selection of neighboring sets has some trade-offs, because it does not ensure about the GEDAR finds the good and opportunistic set of neighboring nodes, the unexpected delays come with minimum throughput.

The depth control routing protocol (DCR) [11] has a similar working nature, as discussed in GEDAR. The major difference between GEDAR and DCR is the moving style of the nodes. The vertical node movement is introduced in DCR to control the topology of the network. To know in advance the greedy approach, the DCR works better where the greedy mechanism of routing fails by using SEA-SWARM architecture. The determination of empty regions is not possible without checking the status of its neighboring nodes, so the main focus of depth control adjustment increases the lifetime and throughput ratio of the network. However, the long route by depth adjustment for neighbors may follow the network in large end-to-end delays.

Void aware pressure routing (VAPR) [12,13] used for monitoring purposes have 4G mechanisms, including space and time. By deploying the on-board pressure gauges at the surface of the ocean, it works directly to send the data packets to the master station. For this, VAPR uses three parameters like hop count, sequence number, and depth information for making the direct path to the closest sonobuoys. The central theme behind the pressure routing is worked in the presence of an empty region, and it is because the location and depth directly control the onboard surface station via an alternate route. Additionally, this scheme increases the network delays when the route is calculated on-board station.

Layer-by-layer angle-based flooding (L2-ABF) is introduced in [6,7] in which every node calculates its flooding angle towards the destination to forward the data packets in the layer hierarchy. The layered approach is used for the deployment of nodes so that there is no need for explicit location information about the nodes. Once the nodes have been deployed, the IDs are assigned that are static throughout the network lifetime. L2-ABF provides a 3D communication zone for its flooding so that both ends must complete the end-to-end delivery.

Another approach AHH-VBF is adaptive hop-by-hop vector-based forwarding (AHH-VBF) [13], in which the radius information instead of angle is used. By using radius information, it quickly reduces the consumption of energy issues because the radius information is done by using acoustic wavelengths. Therefore, there is no need to take extra calculations between source and destination. This scheme enhances the rapid data packet forwarding ratio, reduces the delay, and makes an energy-efficient scheme.

For the selection of nodes and their route towards the destination, the hop-by-hop dynamic addressing-based routing protocol for pipeline monitoring is discussed (H2-DARPPM) [14]. The parametric value for the selection of nodes is energy consumption. It uses the dynamic addressing scheme to each node address so that the PDR is increased, but this approach is costly for network topology.

In [15], the vector-based void avoidance (VBVA) works for mobile underwater sensor networks to address the void routing problem. It works with two novel features vector shift and back pressure to handle the void empty regions. For the empty regions that occur in the boundary of the network, the vector shift is used to deal with these regions; meanwhile, bask pressure is used to avoid the concave empty regions. The primary strategy is that it does not need any prior information about the network due to VBVA’s work with an on-demand approach. It is the first void empty region protocol that works with 3-dimensional topology with mobile nodes. The simulation results show that the PDR is achieved and minimizes the Packet Lose Ratio (PLR).

An energy-efficient watchman-based algorithm (EE-WBA) [16] uses a novel node called watchman node that applies in a distributed based network for detection and avoidance of empty regions near the sink neighboring nodes. Those nodes that died early due to high deprecation of the bottom nodes can rescue with the help of watchman nodes. Watchman nodes are dedicated nodes that deploy for this task. The data are transfer successfully from dying nodes to watchman nodes and the responsibility of the data storage to the sink comes from watchman nodes.

Another article in which introduced the approach for reliable data delivery underwater. The neighbor node approaches distinct energy efficient mates (NADEEM) [17] with two invariants like fallback and transmission. Both these are following the greedy approach to forwarding the data among the nodes of the network. The neighboring node is only eligible to send the data when the route towards the destination is not avoided. Five parameters are considered for this work like PDR, PLR, energy consumption, throughput, and a fraction of void nodes for simulation. The proposed technique performed better than the baseline approach GEDAR [9] in terms of void nodes and corresponding consumption of energy. The major limitation of this work, the route is not every time clear with respect to void nodes, so most of the time, NADEEM does not detect the void nodes.

## 3. Problem Explanation

The underwater topology is randomly deployed where the source and sonobuoys are far apart from each other. Therefore, the energy consumption of nodes is deprecated. Forwarding nodes try to transmit the data, but early deprecation of nodes (that bear the total traffic of the source and intermediate nodes) creates empty regions. Furthermore, when the node starts data forwarding and does not know the exact location where empty regions exist [38]. Mostly, the data are not forwarded due to the presence of empty regions inside the route information. The proper angle towards the node does not consider while forwarding; as a result, the delay and low PDR come into exists. The empty gaps near the sink’s neighboring nodes are needed to fix, as these sink neighboring nodes are responsible for covering these void holes successfully at both ends. However, the empty regions can also be covered by the adjustment of nodes depth level. The adjustment of the node’s depth is based on angle and movement as described in Figure 1. The movement and alignment of nodes are discussed in the literature and in the baseline approach that is taken for comparison, i.e., angle-based approach where each node is aligned up to some angle and with layer-based hierarchy. Moreover, the overhead cache is increased when, for each iteration, the angle needs to be computed. Another problem is that by checking the continuous energy status of nodes, like sink neighboring nodes, is not possible as per some opportunistic routing protocols do this easily [39]. Additionally, most of the previous approach where these issues try to enhance the energy of sink neighboring nodes, is all in vain. The reason is that the energy of the overall network is decreased when a large amount of this material is considered to be dedicated to particular sink neighboring nodes in the underwater acoustic environment. Therefore, the energy sources our primary check for the proposed technique. This approach is also successful in sparse and dense water areas.

For the problems mentioned above, therefore, we need an energy-efficient technique for like TANVEER with its three subsections like LBA-TANVEER, DPD-TANVEER, and BIN-TANVEER. The LBA is used to control the empty region’s occurrence by using the location of the node and its energy status, while for reliable data delivery the DPD-TANVEER works out. To rescue the data of empty regions before and after the node’s deprecation, the BIN-TANVEER is used. Furthermore, the detail of the proposed system model is presented in the next section.

## 4. Proposed Model

The proposed system model consists of multi-sink architecture that provides communication with all IoT enabled sensor nodes [40]. Nodes are not randomly deployed but in a novel layer-by-layer hierarchy in which every node obtains a unique ID that is static unless the communication is not started. These nodes are responsible for collecting and transmitting the data to each other and send them to the sink of neighboring nodes. Sink neighboring nodes further send to sonobuoys that are present at the surface of the water. Some nodes are placed at the bottom of the ocean that are usually called anchored nodes. The purpose of adjustment of nodes in a layer-by-layer manner is basically to support the dynamic environment of water, as water is a very remote and shallow area, and it is believed that the unmanned effort is required so it is necessary to set the criteria to control these nodes random movements that continuously happened from water currents [6,7]. By assigning the IDs and its mechanism we introduce the LBA-TANVEER in which the whole mechanism is presented.

The medium of communication between anchored and sonobuoys is the acoustic channel, as this is the practice of all ASANs. At the terrestrial environment, the sonobuoys used an audio signal to send the data to the base station further [41]. It has been assumed that packets received at any sink would be consider successfully received at all sinks by using the edge of the multi-sink model. For example, if the node ‘*i*’ sends data to another node ‘*j*’ with energy ‘*E*’ and vice versa, it first calculates the angle by using the tri-angular approach and sends the data accordingly, and then the consumption of energy at this event is the same at both ends.

In our work, we made some consideration of this and it is because our focus was on the energy efficiency of the model and avoidance of empty regions [42,43]. (i) The first and foremost consideration is that to sends data for one node to another, it first calculates the angle coordinates on the base of the vector localization service [26,40,41,42,43,44]. (ii) All ordinary nodes send the data packet to sonobuoys simultaneously, and the sink node can receive multiple packets at the same time without any loss of data and collision. (iii) The vertical movements of nodes are negligible; by the way, the horizontal movement of nodes is considered with water current [45,46]. (iv) All the nodes have unique IDs assigned by LBA and will not change in the rest of the network lifetime. (v) The BINs are deployed near sink neighboring nodes so that loss of data inside the empty regions are recovered. (vi) The finding of empty regions is usually near to sink neighboring nodes so that the BIN is also deployed near the sink zone. Figure 2 presents the proposed model in which anchored nodes deployed at bottom that initiates the communication through relay nodes with a mixture of founding empty nodes by using tri-angular calculation. The layer adjustments start from bottom to top so that the communication starts from the first sent by anchored (bottom) and received at the end by sink nodes (top).

## 5. The Tri-Angular Nearest Vector-Based Energy Efficient Routing (TANVEER)

To ensure reliable data delivery between source and sonobuoys, TANVEER considers three basic parameters for forwarding the data among the nodes such as angle, energy, and number of nodes. These three parameters play a part and parcel role to implement the said idea. These parameters improve the performance of the algorithm and make it energy efficient. The occurrence of empty regions is usually found around the sink neighboring nodes. Three types of formats are present for nodes communication as horizontal, vertical, and diagonal according to the availability of the next node. Firstly, all the nodes are assigned in a layer-based adjustment in which each corresponding upward and downward layer have equal distance. Data are delivered through the angle base approach. Basically, every node computes the angle three times in all possible directions and moves forward for the next node. The data delivery and recovery are only performed on the decided routing path; furthermore, for the recovery process, the binary inter-nodes are introduced where the angle does not make it possible to compute. In order to minimize collision and loss of data packet, one node transmits a one data packet at a time. The detail of each phase is described in the following subsections.

### 5.1. Empty Regions Selection

An optimal way to select the empty regions is based upon the number of resources used in the acoustic environment. The energy is one of the most fundamental resources where nodes are enabled to join the network and process the data communication towards the sonobuoys [47,48,49]. The section of empty regions ‘*Ser*’ is directly based upon the node’s energy ‘*En*’, where ‘*i*’ is the total number of empty regions selected (i varies from 0 to *N*; where ‘*N*’ is the total number of nodes inside the empty regions) every node, before forwarding the data packets computes angle three times for three directions like (horizontal ‘x’, vertical ‘y’, and diagonal ‘z’). Multiple neighbor’s nodes with respect to angle are also selected when there is no empty region throughout the routing path from the anchored node to sonobuoys. Additionally, the related mathematical equation is used to selection of total number of empty regions as in Equation (1):

(1) $$S_{er}=\left(En/\left(Ni\;*\;A\;\left(x,\;y,\;z\right)+1\right)\right)\;$$

This equation boosts the concept of smooth delivery of data (when there is no void nodes [50,51,52]) and trace out the empty region on the routing path. It fits for medium and large-scale networks because when the number of nodes increases, the ratio of occurrence of empty regions ‘*Er*’ also increases [53,54]. Selection of the multiple number of nodes increased the data packet ratio and throughput of the network. Another factor that needs to be discussed is the packet acknowledgement (*P_ACK_*) that helps to select the next layer and node, once the layer will be selected, that is obviously select by *P_ACK_*, it is easily select the next data forwarder node. This selection leads the routing path through bypassing the empty regions [40] until the data are received to sonobuoys. Basically, the *P_ACK_* is defined as and is the confirmation of the receiving of beacon or hello packet message. It is the practice of acoustic communication before sending the data to check which nodes are alive/part of the network [55]. If the packet is sent by the node of the first layer (*N* (*l*_1_) to the node of the second layer (*N* (*l*_2_) with the difference between layer (*D*), it first computes the angle with all coordinates to create an existing routing path between these layers with a possible direction. The selection of angle direction would be considered, where there is no ‘*Er*’ throughout the path. If the consecutive layers are selected for forwarding the data, all layers are responsible for providing their number of nodes to make the route robust. For example, the path was found to be smooth from anchored node to the first layer, and ‘*Er*’ occurs in the sink neighboring nodes area. The corresponding angle changes the direction of the vector for the flooding of data. The route vector can change at any stage by avoiding the ‘*Er*’ occurrence, as this is only done to handle the exceptional case. The corresponding mathematical Equation (2) below describes the selection of layers and nodes and routing path with angle:

(2) Ni={ (N (l1)^(N (l2)∈P_ACK : ∃ N (Ax, Ay, Az)→Sn 

where ‘Sn*’ is the closest optimized sink in which the total number of nodes with the difference of empty regions that selects for data delivery as in Equation (3):

(3) $$Sn*=opt\;\sum_{i}^{n}Min\left\{\;Tn-Er\right\}\;$$

### 5.2. Energy Efficient Nearest Vector Selection

Nearest vector selection is based on the number of nodes and angle calculation, as discussed earlier. The angle will be calculated in three consecutive adjacent directions. The movement of the angle vector is only selected when there is the next forwarder node, and its neighbor node is available without any empty zone. The next forwarder node further computes the angle again and repeat the aforementioned process in order to avoid the empty nodes. Energy is an essential part of underwater communication, especially in the IoT enabled environment [56]. Due to acoustic communication, the nodes are deprecated their energy value continuously. Furthermore, mobile water and dynamic topology also have an effect on the overall health of the network. As the path becomes longer by selecting the number of nodes towards the sonobuoys, then the nearest vector path is selected. It is because; by computing the angle thrice only those nodes are selected that cover the angled cone and have the next link with forwarder nodes Moreover, a detailed survey of underwater ad hoc networks is presented in [46,47,48,49] where various methods of underwater communication are discussed. For every nearest vector it selects according to the availability of the node ‘*Nv*’, and angle coordinates ‘*A*(*x, y, z*)’, in such a way that ‘*Nv* *∈ A* (*x, y, z*)’ but not empty regions ‘*Nv* ∄ *Er*’ towards the ‘*Sn*’, Equation (4) becomes:

(4) $$Sn=Nv*A\;\left(xyz\right)\;\cup \;Nv\;∄\;Er\;\leftarrow P_{\mathrm{ACK}}\;$$

After the selection of nearest vectors from the set of nodes, the routing path is established towards the sonobuoys, near to it, if the sudden node dies and is able to create the ‘*Er*’, the routing path is changed to ‘*P_CHG_*’ and calculates the angle once again. The ‘*P_CHG_*_’_ mechanism is defined as it is the alternate adjustment for routing path while the next forwarder acknowledges even received the ‘*Nv*’ nodes, and its frequency ‘*f*’ is the proper set of ‘*Tn*’. If ‘*Tn*’ is less than ‘*Nv*’, the ‘*Sn*’ directly available to receive the packet such that ‘*Nv* *∝* *f*’ and according to the path selection mechanism, so the difference ‘*D*’ between the nearest sonobuoys and the node is basically as in Equation (5):

(5) $$D\;(Sn)=(N(l_{n}-l_{1})\to \varphi \;\left(fn\cap Tn\right)$$

*P_CHG_* will be implemented through the expression as in Equation (6):

(6) $$P_{CHG}=Pack\;↔Sn\;:\;\exists \;\sum_{n}^{0}minNv*\forall \;f(Nv*(1-Tn\;\oint_{i=0}^{n}fn(Er\frac{\pi }{2}*\left(A\;\left(x,\;y,\;z\right)\right)$$

It is a bi-conditional structure that means the path does not change if and only if the ‘*ACK*’ is continuously received with sonobuoys ‘*Sn*’. The available nodes from ‘*0 to Nv*’ the minimum selection of total frequency of nodes ‘*f*’ is subtracted with 1 from the total number of nodes by calculating the angle coordinates to change the path ‘*P_CHG_*’. Ultimately it sets the path change, and the route again receives the packet from the node (1 is used for this node that holds the data packet before getting the node of the empty region). Meanwhile, by changing the path, it has the opportunity to select more nodes to forward the data packet further; the detail of this strategy is discussed in Algorithms 1 and 2. Additionally, if the nodes cannot be selected (or there is no availability) at the top of the layer near to water surface, the packet is discarded, and the alternate novel approach is used as discussed in Algorithm 3, in which extra watchman nodes are deployed around the edges and near the sonobuoys to rescue the discarded data packets and minimize the occurrence of packet loss ratio.

## 6. Layer-Based Adjustment (LBA)-TANVEER

Most of the experiments have been done in deep and shallow water deploying nodes for different purposes such as oil exploration and mineral findings and for some safety-critical applications like [43,44,45,46,47,50,53]. When the environment is enough complex, then the deployment of nodes in random order is not the rational decision; therefore, to avoid as well as accurate detection of empty regions between the nodes the layer-based adjustment of the nodes is aligned for our experiment. All the nodes are deployed in equal layers with multi-sink architecture in such a way that every layer assigned with a unique ‘*ID*’. Each sensor node gets a layer ‘*ID*’ by broadcasting the ‘*Hello Packet* (*HP*)’ from the sonobuoys. As all the sensor nodes are below the sonobuoys, so it is easy to attempt the layer ‘*ID*’ so that each node will be uniquely identified by receiving the ‘*HP*’. In order for each rebroadcasting mechanism, the decrement is applied by the total layer count field of the ‘*HP*’. The numbering of the ‘*layer-ID*’ is assigned in ascending order from top (sonobuoys) to bottom (anchored nodes) so that the first layer is the closest layer towards sonobuoys and so on, and this is continued until the whole nodes of the network are assigned a ‘*layer-ID*’.

Initially, the ‘*layer-ID*’ will be static until the sonobuoys send the next update after a specific time interval. The nodes that directly receive the ‘*HP*’ from sonobuoys will be considered the first layer, and with the decrement of one in the total layer, count field rebroadcast the packet for other below nodes of the first layer. Hence the second decrement is for assigning the ‘*ID*’ for the second layer nodes until the total layer count field becomes zero. As the counter field becomes zero, the further broadcasting of ‘*HP*’ is stopped. All sonobuoys that usually present on the surface of the water have fixed static ‘*layer-ID*’ that is ‘0′. Broadcasting is started with the total layer count field value that is ‘9′, and the ordinary nodes that is ‘*N*_1_ *to N*_6*′*_ received the ‘*HP*’ directly from the sonobuoys and were assigned the ‘*layer-ID* ‘*1′*. with the decrement of 1 the nodes ‘*N*_7_ to *N*_12_*′* received ‘*HP*’ from the upper layer nodes and were assigned the ‘*layer-ID* ‘*2’’*.

Meanwhile, this will continue until the nodes of the last layer get ‘*ID*’. In case the nodes received multiple ‘*HP*’, the Tri-angular nearest vector approach is used to compare the value of the ‘*Max-Layer-count*’ field of ‘*HP*’. If the field value is the same, take one ‘*HP*’ into account and discard the other ‘*HP*’, and with different ‘*Max-Layer-count*’ field values, the nodes chose only the packets with greater value in the field and discarded other packets. Figure 3 presents the overall scenario of LBA-TANVEER, and Algorithm 1 discusses the assigning of layers ‘*ID*’ in LBA.

**Algorithm 1:** Layer-Based Adjustment of Nodes.1. **Procedure**
 ASSIGNING LAYERS (Source, Sonobuoys (*Sn*)) 2. Total Layers (*TL*) = 9 3. **IF** HP: Hello Packet notification having seq _Layer-ID_
4.  ***If***
*Own-Layer-ID* = 00 5.   ***For***
*n* = 1 6.     ***if***
*n*
∈N && s∈Sn then
7.     HP. Coordinates ←N (x, y, z)
8.     *HP. Add (seq _Layer-ID_ (N ID (x), (y), (z)))*
9.       For (N (l1)^(N (l2)∈PACK do
10.       Assigning _Layer-ID_
11.    **end if**
12.      **End For**
13.       ***IF***
*(Own-Layer-ID ≤ n)* 14.          *Discard HP*
15.         ***Else***
16.          *Own-Layer-ID = n*
17.          *n* + + 18.          *Broadcast HP further*
19.       ***End IF*** 20. ***End IF*** 21. ***End If*** 22. ***End for*** 23. *No further broadcast for this Sink-HP* 24. ***End Procedure***

## 7. Data Packet Delivery (DPD)-TANVEER

Any sensor and actor nodes want to begin the transmission; firstly, they check the remaining energy values ‘*Re*’ of the corresponding nodes and calculate the angle for flooding data packets. ‘*P_ACK_*’ is already defined in such a way that the data packet is only sent when the next forwarder nodes are available to receive the data as well as sends the proper acknowledgments. The angle calculation is based on three attempts as the horizontal with the same layer nodes, vertical for the upper layer and diagonal to support horizontal and vertical communication. The nearest vector is only calculated in order to respond to the tri-angle strategy in such a way that which path is best for forwarding the data packet throughout the sonobuoys. The adjustments of layers are made during packet acknowledgment via Equation (2) and the shortest path without considering the empty regions of Equation (3) play our role. If the forwarder layer does not receive acknowledgment due to empty regions, the path change mechanism will be applying according to Equation (6) in which the path will only be changed when ‘*Er*’ is present with the neighboring node where the data are being received by the sonobuoys. Another reason for path change is also described if the next forwarder node is not available for another node, then it automatically considers the packet to be discarded. Algorithm 2 describes the data packet forwarding towards the sonobuoys via the nearest vector-based node’s availability. In this algorithm the node ‘*n_i_*’ want to send the packet node ‘*n_j_*’ with the distance ‘*D_ij_*’ from ‘*l*_1_ *to l*_2*′*_ without considering the ‘*Er*’, the total frequency of nodes ‘*fn*’ is basically from source to sonobuoys is, ‘*Tfn* = *D*(*n_i_, l*_1_) ∗ *D*(*n_j_, l*_2_) − *P* (*Er*)’, where ‘*Sn*’ is the total number of nodes available in each layer to find the closest next forwarding nodes with short distance as defined in Equation (5). In order to define the flooding zone, we are assuming that by using the basic linear formula ‘*θ* = 90 ± 10 *K*’ the nodes have the capability of computing the angle. Here, ‘*K*’ is a variable and has a finite set of values, ‘*K* {1, 2, 3}’.

**Algorithm 2:** Angle Based-Data Packet delivery.1. **Procedure** ACQUIRE ANGLE FOR NEXT NODE (Source_ni_, Sn (P_ACK_) 2. **For**
Re ∈Ni do
3.   *Tf_n_ = D(n_i_, l_1_) ∗ D(n_j_, l_2_) − P (Er) **then***
4.   *θ = A (x, y, z)* 5.   *Adjust angle for corresponding nodes ni → nj*
6. ***End For*** 7. *n*
←1 8. *j*
←1
9.    ***While***
*θ = 90 ± 3K*
**do**
10.    Pack ↔Sn ∪ Sn=Nv∗A (xyz) ∪ Nv ∄ Er ←P Ack 11. **If** Pack ∄ Ni and D (Sn) = (N (l_n_-l_1_) $\to \varphi \;\left(fn\cap^{\;}Tn\right)$
**then**
12.   P_CHG_ =$\;\sum_{n}^{0}minNv*\forall \;f(Nv*(1-Tn\;\oint_{i=0}^{n}fn(Er\frac{\pi }{2}*\left(A\;\left(x,\;y,\;z\right)\right)$ 13. **For** Ni ←ni−D (Sn)
14.   Nj ←nj−D (Sn)
15.    **End While**
16. **End For** 17. **Else**
18.    **Modify**
*θ*
*= A* (*x, y, z*) ↔P*_CHG_*
→ 0 < θ < π 19. **End if** 20. **For**
*Er*
**do**
21.    **for** Re (Ni →Nj)
**then** 22.    Compute distance for nearest neighbor vector node **then**
23.       **if** Nv: ∀
*Tf_n_ = D(n_i_, l_1_) ∗ D(n_j_, l_2_) − P (Er)*
***do***
24.       Dissemination of data packet delivery until Er ↔ P_CHG_
25.       **end if**
26.     **end for** 27. **End For** 28. **End if**
29. **End Procedure**

After this, if there is no outcome in terms of packet receiving the source node this will increase the value of ‘*K*’, as the value of ‘*K*’ is increased the flooding zone is also increased under the angle condition that is (*0 < θ < π*). The value of ‘*K*’ is helpful to control the amount of energy consumption of the said node and end-to-end delay. The nodes movement directly depends on the base of the decided value of ‘*K*’. In the case of multiple nodes inside the zone, all nodes will calculate their priorities and send ‘*HP*’ to the source node, and the data are forwarded by highest priority node. The priority queue that is considered is of maximum 5 that will save the source node information, the advantage of this strategy is the next time data that will be sent without calculating angle zone, but this will only be kept in a specific interval of time to maintain the priority acknowledgment, otherwise it will again compute the appropriate angle. Usually, for the medium and small-scale network, this would not happen, and once the angle has been computed, it will remain the same and be used for the rest of the network lifetime. Hence, every sensor node knows its base angle that is ‘*π/2′* in the upward, horizontal and diagonal direction and it is also a built-in hardware module; by the way, according to the environmental situation every node have enough capability to compute and increasing the flooding zone by changing the value of ‘*K*’. Here it is important to know that on every attempt the previously calculated area will be added into the newly defined zone to take advantage of mobility. Further, Figure 4 presents the computed angle with the value of ‘*K*’, and Algorithm 2 discuss the angle-based data packet delivery, i.e.; DPD-TANVEER, while tri-angular adjustment of horizontal ‘*A*(*x*)’, vertical ‘*A*(*y*)’ and diagonal ‘*A*(*z*)’ and the scenario of DPD-TANVEER are discussed in Figure 5 and Figure 6, respectively.

## 8. Binary Inter Nodes (BIN)-TANVEER

As the data packet delivery is increased, the probability of the empty regions inside the network is also increased [45,46,57]. Therefore, the BIN-TANVEER is used to ensure the packets that are not delivered to the next nodes due to empty regions is must be delivered to the desired destination via the path change mechanism. For this, we introduced ‘Binary Inter Nodes’ called watchman nodes inside each layer. These watchman nodes are responsible for controlling the communication at both ends, like the left and right of the network. Figure 7 illustrates the proper mechanism of BIN-TANVEER. For example, the source node ‘*S*’ wants to send the data packet to the nearest nodes, and it first obtains the information of the above two layers with all corresponding neighbor nodes. Up to two layers, those nodes that are closer to each other are ‘*n*_1_*, n*_2_*, n*_3_*′*, and ‘*n*_4_*′*. In the first layer, the ‘*n*_2_*′* node is nearest with respect to energy, and TANVEER further explores to the next node to ‘*n*_2_ *like n*_3_ *and n*_4_*′* for other next two layers. The angle is computed for the best three possible directions towards the destination with the value of ‘*k*’, as discussed in Algorithm 2. As ‘*n*_4_*′* shows the empty region, it moves toward the nearest node in a relevant direction with a reference of the calculated angle. The possible routes of movements are horizontal with the same layer or vertical, and the diagonal path is available for the next upper layer according to the availability of the nodes. If the data are delivered and *‘n_4_′* becomes an empty node, then BIN-TANVEER works.

It takes the data from the empty node like ‘*n*_4_*′* and delivers them to the next available node. The recovery procedure works with looking forward to the least number of layer count and tries to find out the next nearest node in order to proposed mechanism energy efficient with all aspects. In this way, instead of dropping the packets and increasing the packet loss ratio of the overall network, BIN nodes are responsible for doing this every time. Therefore, they come into contact or active only when the empty regions are detected. Meanwhile, the detection of empty regions is done by receiving the ‘*P_ACK_*’ of the nodes as in Equation (6), and ‘*P_CHG_*’ is basically the function of BIN-TANVEER, and it used for the recovery procedure. In an exceptional case, if the availability of the node is not present around the empty regions, then BIN sends a data packet greedily to sonobuoys. Otherwise, it transfers the information to the nearest node for successful data communication among the network nodes. Figure 7 shows the working of BIN-TANVEER and Figure 8 represents the transmission adjustment of watchman, while Algorithm 3 depicts the proposed mechanism. Moreover, the flowchart of proposed scheme and its sub-sections are illustrated in Figure 9.

**Algorithm 3:** Empty Regions Recovery Using Binary Inter-Nodes.1. **Procedure FORWARD THE DATA PACKETS (*θ*) (Source, Sonobuoys)** 2. **IF Empty region or node = 0 then**
3.   Compute the angle with A (x, y, z) 4.   Select the appropriate nodes 5.   Forward the data packets () 6. **ELSE**
7.      **For** Nv: ∀ *Tf_n_ = D*(*n_i_, l*_1_) *∗ D*(*n_j_, l*_2_) ←
*P (Er)*
***do*** 8.      **Modify**
*θ*
*= A* (*x, y, z*) ↔P*_CHG_*
→
*0 < θ < π*
9.     ***If***
Pack ↔Sn ∪ Sn=Nv∗A (xyz) ∪ Nv ∄ Er ←P Ack 10.    then
11.    Re-schedule angle and forwards the data packets () 12.    **Proposed mechanism ()** 13. **END IF**
14.      **End For**
15.     **End If** 16. **End Procedure**

## 9. Achievable Regions

**Figure 11:**
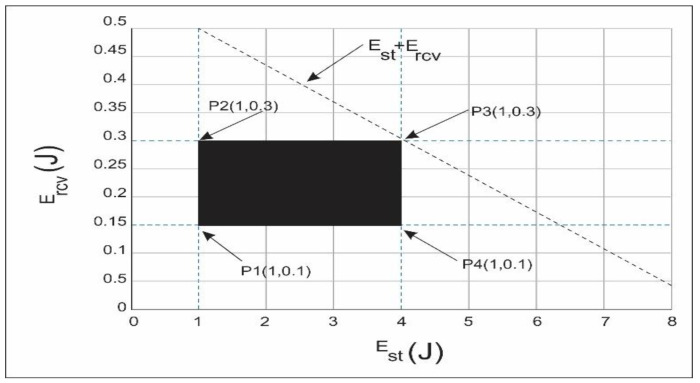
Possible feasible region of energy minimization.

In order to calculate the achievable regions inside the network in an optimized manner, we used a linear programming approach in this section. To obtain the optimal result, the mathematical technique linear programming is used as same as [17]. The objective function that we analyzed through linear programming, minimum energy consumption, and maximum throughput is discussed in Figure 10 and Figure 11.

### 9.1. Minimization of Energy Consumed

To achieve an optimized result to calculate the energy consumed, the linear-based functions and constraints are the best way starting from the objective function and followed by linear constraints. The objective function of the minimization of energy consumed is defined as:

(7) $$\sum_{n=0}^{max}min\;({\mathrm{Energy}}_{\mathrm{con}}\;(n))\;\forall n\;\in \;n_{\max}\;$$

The linear function of energy consumed minimum is given in Equations (8)–(10).

(8) $$F_{1}:\;E_{st},\;E_{end}\leq E_{I}$$

(9) $$F_{2}:\;N_{L}\;\leq \;N_{l}^{min}$$

(10) $$F_{3}:\;C_{r}\;\leq \;C_{rmax}$$

Equation (8) deals with the required energy to start the transmission and consumption should be less than the initial amount of energy of the node. Equation (9) discusses the next layer node that has the minimum energy consumption. Equation (10) is about the transmission range with its maximum transmission range of the node. The proposed scheme of calculation of the energy consumed per node is in Equation (11) as follows where energy consumed includes both starting and ending value of energy per node i.e.,:

(11) $${{Energy}_{consumed}}_{\;}(n)=\sum_{n=0}^{max}min\;({Energy}_{con}(n))\;\forall n\;\in \;n_{\max}\;$$

(12) $${{Energy}_{consumed}}_{\;}(n)=E_{st}+E_{end}\;\forall n\;\in \;n_{\max}$$

where,

(13) $$E_{st}=P_{tx}\frac{Data\;size\;\left(n\right)}{Data\;rate\left(n\right)}$$

‘*E_st_*’ is the amount of energy when the transmission is started and, ‘*P_tx_*’ is the transmission power.

(14) $$E_{end}=P_{rcv}\frac{Data\;size\;\left(n\right)}{Data\;rate\left(n\right)}$$

‘*E_end_*’ is the amount of energy when the transmission is ended, while ‘*P_rcv_*’ is the transmission power.

**Graphical expression:** Graphical analysis is present in order to clear the picture of the feasible empty regions. For example, data size = 100 bytes, data rate = 60 kbps, *P_tx_* = {0.5,1, 1.5, 2}W and *P_rcv_* = {0.015,0.025, 0.05,…..,0.1}W, then feasible region for energy minimization is computed as per the aforementioned constraints in Equations (8)–(10):

(15) $$1\;\leq \;E_{st}\;\leq \;4$$

(16) $$0.1\;\leq \;E_{rcv}\;\leq \;0.3$$

(17) $$1.1\;\leq \;E_{st}\;+\;E_{rcv}\;\leq \;0.43$$

The boundary of the feasible region can be plotted in Figure 10 from the above equations:

*P*1 (1, 0.1) = 1.1 J

*P*2 (1, 0.3) = 1.3 J

*P*3 (4, 0.3) = 4.3 J

*P*4 (4, 0.1) = 4.1 J

Hence by selecting any values from these plotting points obtain the available minimization of energy consumption.

### 9.2. Maximum of Network Throughput

As network throughput is the major parameter to determine the efficiency of the network. To improve the network throughput same linear programming approach is used as discussed in energy minimization. The related expression is as follows in Equation (18):

(18) $$\sum_{n=0}^{max}Maximum\;({\mathrm{Thr}}_{\;}(n))\;\forall n\;\in \;n_{\max}\;$$

The following constraints are as follows:

(19) $$C_{1}:\;E_{st},\;E_{end}\leq E_{I}$$

(20) $$C_{2}:\;N_{L}\leq \;N_{l}^{min}$$

(21) $$C_{3}:\;C_{r}\;\leq \;C_{rmax}$$

(22) $$C_{4}:\;D(n_{i},\;l_{1})\leq \;D(n_{j},\;l_{2})$$

(23) $$C_{5}:Min\sum_{n=1}^{max}B_{frw}^{n}$$

Equation (19) deals the required energy to start the transmission, and consumption should be less than the initial amount of energy of the node and Equation (20) discussed the next layer node that has the minimum energy consumption. Equation (21) is about the transmission range with its maximum transmission range of the node. Equation (22) shows that the distance between two nodes ‘*i*’ and ‘*j*’ is minimum for successful communication. The proposed scheme of calculation of the maximum throughput is the bandwidth assigned for the next forwarder node in the case of empty regions and is in Equation (23) such that ‘*B_frw^n*’ and for non-forwarding node is ‘*B_*(*N-frw*)*^n*’. The overall bandwidth is calculated for the aforementioned equations are below where bandwidth is assigned for 150–300 KHz as from [17].

(24) $$50\;\leq \;B_{frw}^{n}\;\leq \;100$$

(25) $$200\;\leq \;B_{N-frw}^{n}\;\leq \;300$$

(26) $$250\;\leq \;B_{frw}^{n}\;+\;B_{N-frw}^{n}\;\leq \;350$$

The boundary of the feasible region can be plotted in Figure 11 from the above equations are:

*P*1 (50, 200) = 250 K Hz

*P*2 (100, 200) = 300 K Hz

*P*3 (50, 300) = 350 K Hz

*P*4 (250, 300) = 550 K Hz

Hence by selecting any values from these plotting points obtain the maximized throughput.

## 10. Simulation Results and Discussion

The proposed scheme TANVEER, LBA-TANVEER, DPD-TANVEER, and BIN-TANVEER was evaluated in NS-3 (with AquaSim Framework) against related parameters like packet delivery ratio, packet loss ratio, the fraction of empty region and network throughput. The energy consumption of the network was also measured. All these parameters were compared with the baseline approach L2-ABF [7]. Additionally, our major focus was on the other two constraints like minimization of energy consumption and maximum traffic throughput to achieve reliable data communication in order to reduce void hole occurrence [41,42,43,44,45,46]. Therefore, the analysis of transmission range and energy per data packet was conducted in the result section.

### 10.1. Performance Control Parameters

The performance control parameters directly affect the performance of the network system like transmission range, data rate, energy deviation, and payload of data packets. The nodes were randomly deployed in the form of the layer with the area of 2000 m × 2000 m × 2000 m. The 600 nodes were used, including 40 sink nodes, and the transmission range varied from 150 m to 400 m. The data rate is 16 to 32 kbps for performance evaluation against baseline approach L2-ABF, and the deviation of energy for empty regions was 32, 64, 128 kbps. The data packet payload was set to 100–200 bytes. The value of energy consumption was *P_t_* = 2 W, *P_r_* = 0.2 W and *P_i_* = 0.02 W for transmission, reception and idle energy of BIN nodes, respectively. Table 2 presents the constraints used in the simulation environment.

### 10.2. Fraction of Empty Regions

The fraction of empty nodes becomes compact, then the empty region is created inside the network so the result would be conducted according to empty regions occurrence and compared with L2-ABF. The heterogeneous relation comes into existence in all approaches like TANVEER and its sub-sections, and as the nodes increase the occurrence ratio of empty regions increased. Firstly, the maximum fraction can be observed with respect to DPD-TANVEER, which is under half of the network nodes.

**Figure 12:**
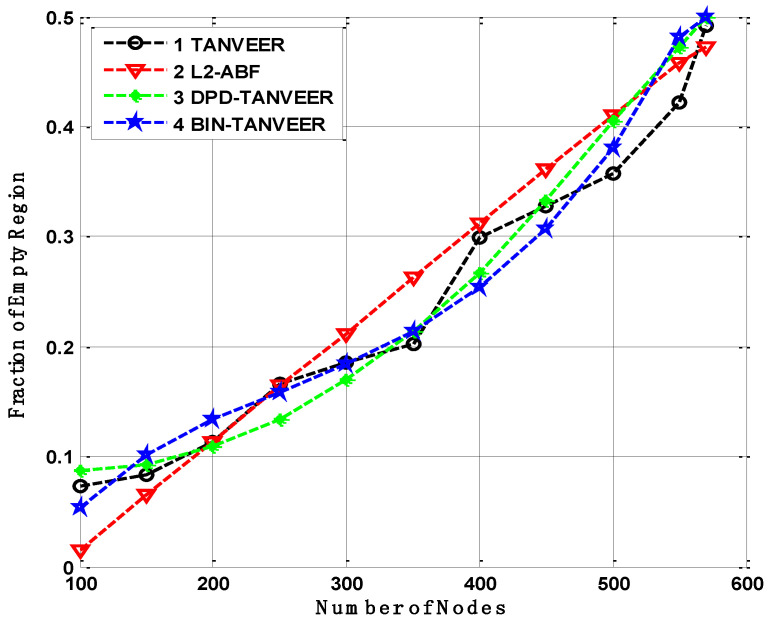
Fraction of empty regions occurrence in TANVEER vs L2-ABF.

**Figure 1:**
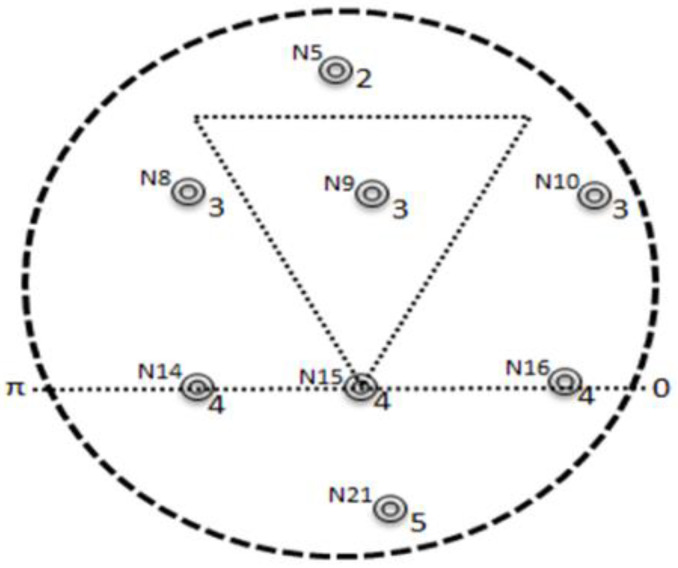
Description of angle adjustment discussed in L2-ABF [7].

**Figure 2:**
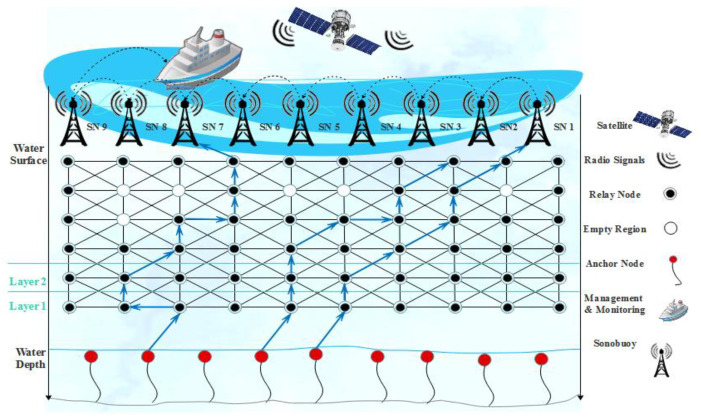
Proposed model of Tri-Angular Nearest Vector-Based Energy Efficient Routing (TANVEER).

**Figure 3:**
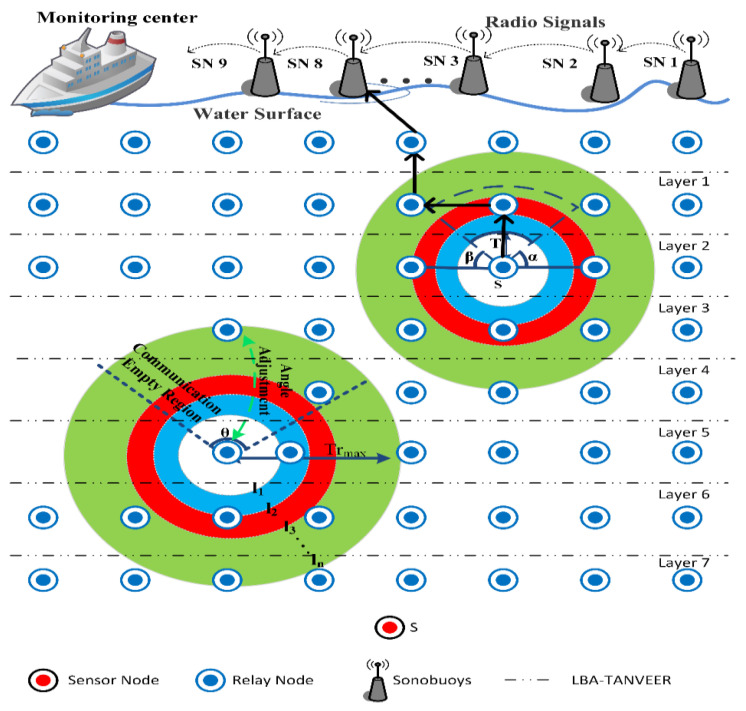
Layers-Based Adjustment (LBA) to ordinary nodes.

**Figure 4:**
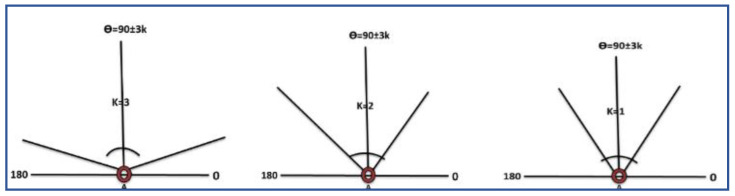
Possibility of angle flooding zone when Node Uses *K* = 1, 2 and 3.

**Figure 5:**
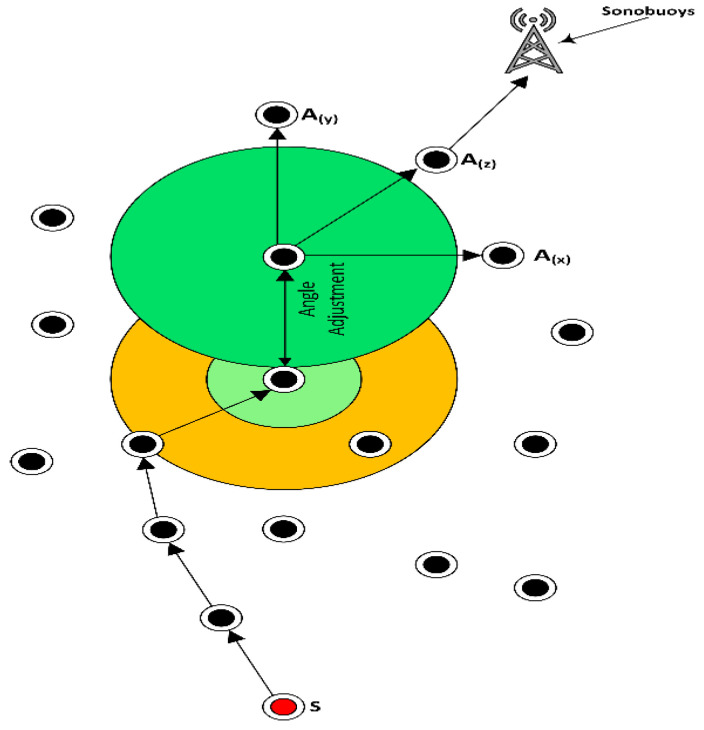
Tri-angular adjustment of horizontal *A(x)*, vertical *A(y)* and diagonal *A(z)* with reference to Figure 4.

**Figure 6:**
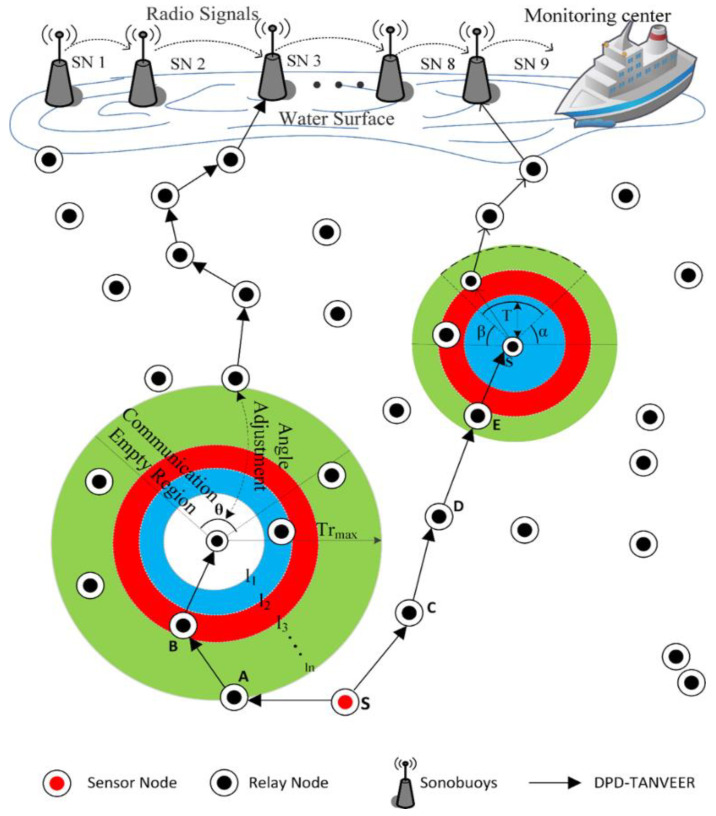
Mechanism of Data Packet Delivery (DPD)-TANVEER.

**Figure 7:**
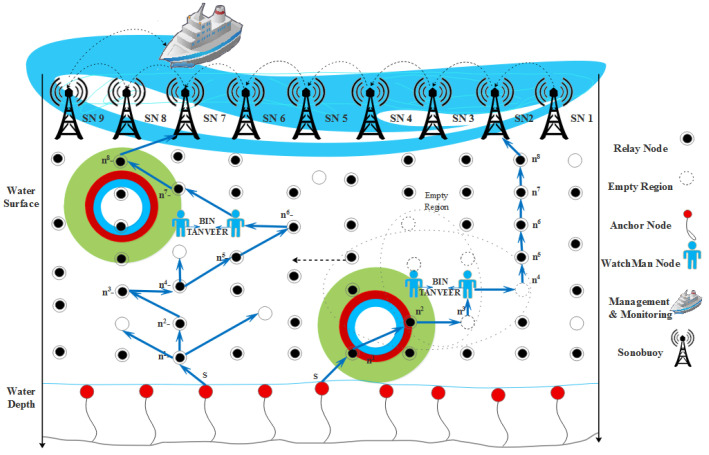
Working mechanism of binary inter nodes (BIN)-TANVEER with watchman nodes.

**Figure 8:**
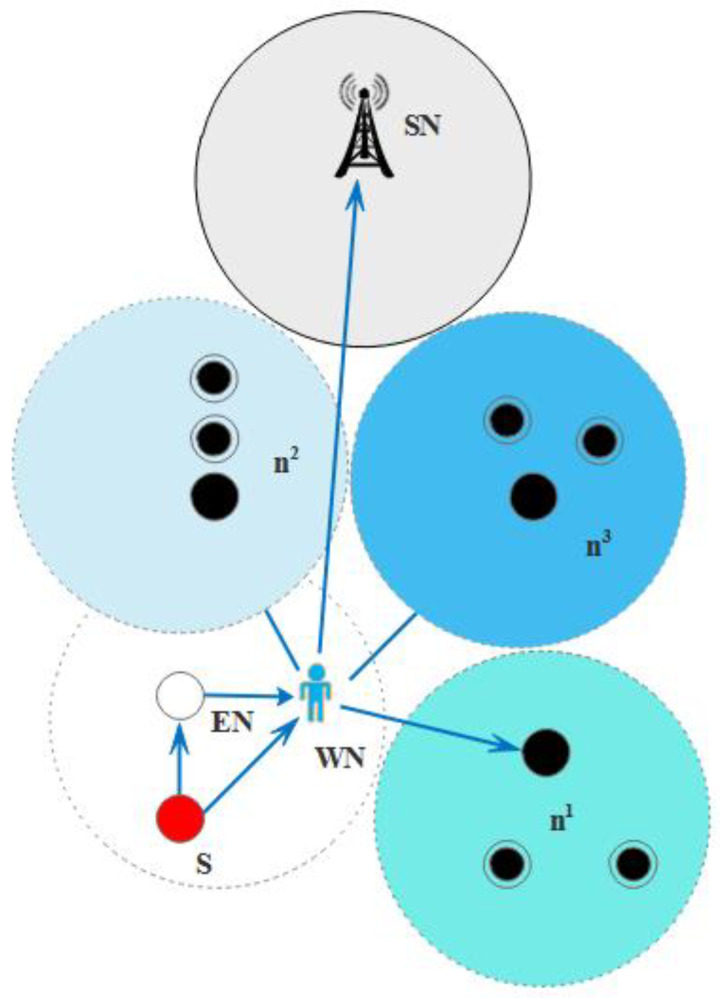
Possible transmission adjustment of Watchman Node (WN) with Empty Node *(EN*).

**Figure 9:**
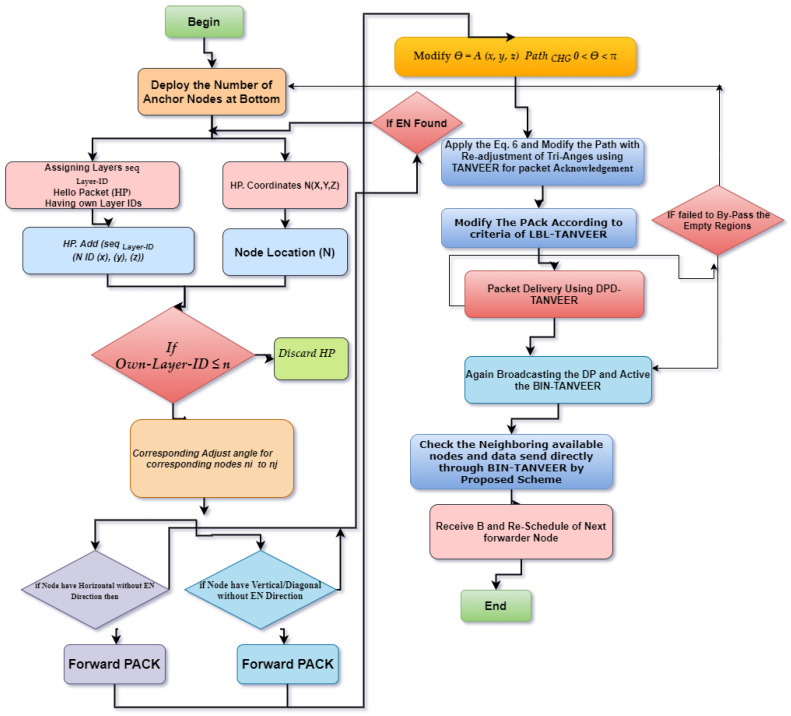
Flowchart of the proposed scheme.

**Figure 10:**
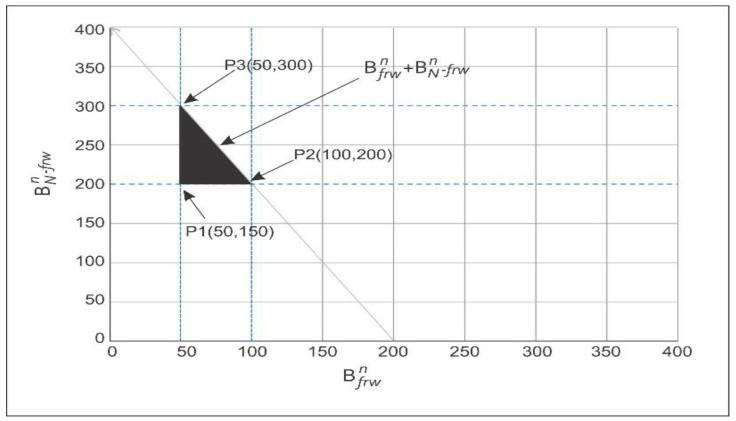
Possible feasible region of throughput maximization.

However, a small upward sliding was observed from 300 to 500 nodes. After this, an increasing trend presents in which a number of nodes remained constant in this area. The decrement is because of DPD-TANVEER when the empty regions are infrequent amount then, for the time being, data are shifted towards the watchman nodes and then again revert to the ordinary nodes. The ordinary nodes further transmit the data to sonobuoys. If there are no further empty nodes, the multiple occurrences of the empty node on the same path is tackled by the help of BIN-TANVEER as watchman nodes. Watchman nodes present in each layer in order to minimize the end-to-end delay. This will help to save not only the time delay but also achieve the purpose of nodes in layers order as it is done in LBA-TANVEER. Both techniques have little similarity in results due to the layer-by-layer angle approach used in L2-ABF and tri-angular approach in TANVEER. The BIN-TANVEER beats the baseline approach as the fraction of empty nodes does not handle by ABF, because of the extra supplementary nodes used like BIN. The direct transmission is sent to the forwarding nodes by calculating the angle, while on the other hand, the DPD-TANVEER handles this situation work with BIN-TANVEER and bypasses the empty regions, as shown in Figure 12.

### 10.3. Packet Delivery Ratio

It is assumed that the delivery ratio is directly proportional to the number of nodes increased in the network [57]. Therefore, the density plays a part and parcel role in the underwater environment. It is clear that the BIN-TANVEER has the highest PDR results due to the functional nature of the proposed mechanism. As BIN is working for the rescue nodes to avoid the data loss, hence the PDR is achieved at the desired amount even in the density of the network. When the trap of the empty region is going to start the avoidance of data loss, alternative nodes in the form of BIN are considered the best substitute to deliver the data to nearby sonobuoys. Meanwhile, the TANVEER describes the average success ratio of packet transmissions, i.e., it is neither high nor low, because it operates is single parameter total packet delivered to the sonobuoys. The dynamic topology of the network is also affecting the efficiency of data transmission due to nodes movements.

**Figure 13:**
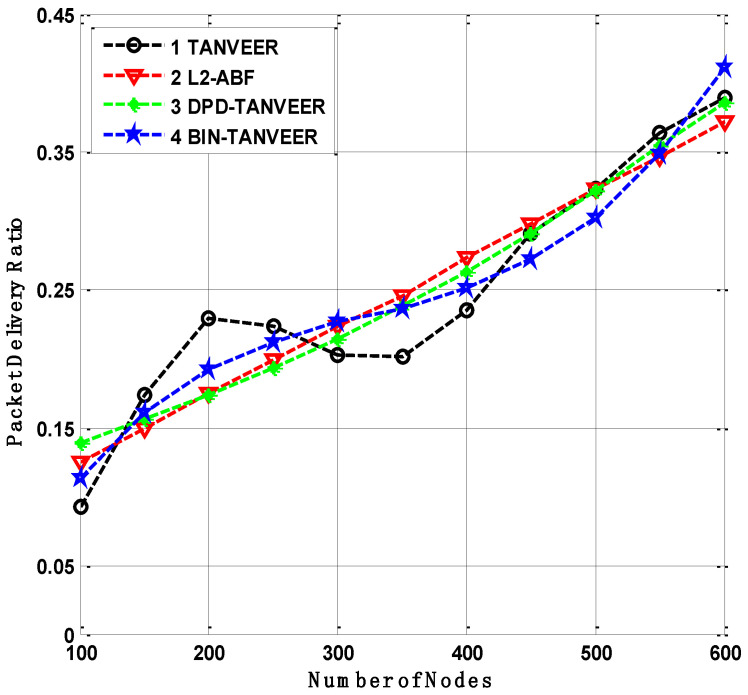
Packet delivery analysis of TANVEER vs. L2-ABF.

As compared to the baseline approach of L2-ABF with the proposed technique is shown in Figure 13. The energy consumption of both techniques is slightly high due to the means of data transmission. The data forwarding in L2-ABF is based on angle cone calculation, whereas TANVEER computed the angle thrice. It also requires more energy for some recovery mechanism. The DPD-TANVEER has achieved the desired results because it considers the only paths that ensure bypassing the empty regions causes high PDR.

### 10.4. Energy Consumption

**Figure 14:**
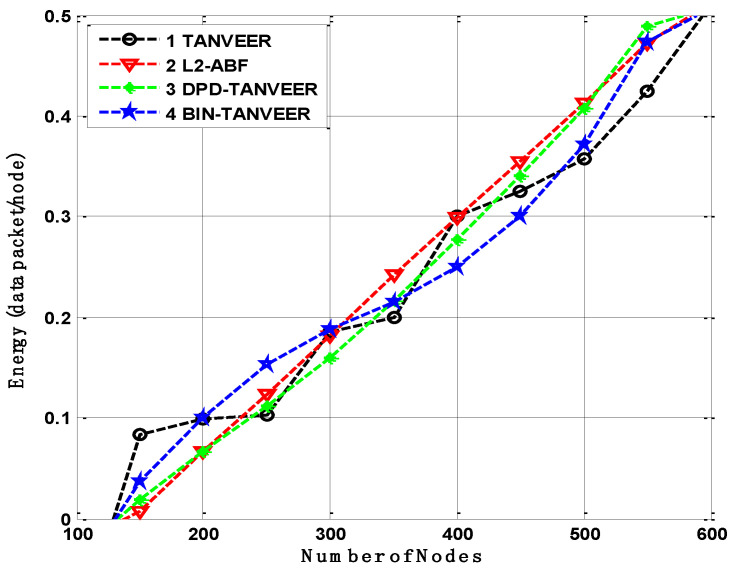
Energy consumption of TANVEER vs L2-ABF.

The comparison of energy consumption is displayed in Figure 14, where the energy is delivering a single packet towards the destination. It observed that the minimum energy consumption for the DPD-TANVEER is when it could bypass the empty regions and directly sends the data packets to the sonobuoys, so it is slightly lower than L2-ABF. The minimum possible energy consumption for each packet is 0.3 J with DPD-TAANVEER and 0.4 J of L2-ABF. This is only due to singe angle-based measuring for the dissemination of the data packets.

The BIN-TANVEER is comparatively higher, saving the amount of energy, as these nodes only come into contact when the empty regions need to be rescued. It is lower than the other two proposed schemes as well as a baseline approach. The foremost reason is the alternative route adjustment for the nearby sonobuoys increase the amount of energy for the time being to perform recovery data from the empty nodes. Nonetheless, this amount is not a sufficient reason for the high consumption of energy requirement as L2-ABF is considered the battery resources comparatively higher than the technique as mentioned above.

### 10.5. Throughput

**Figure 15:**
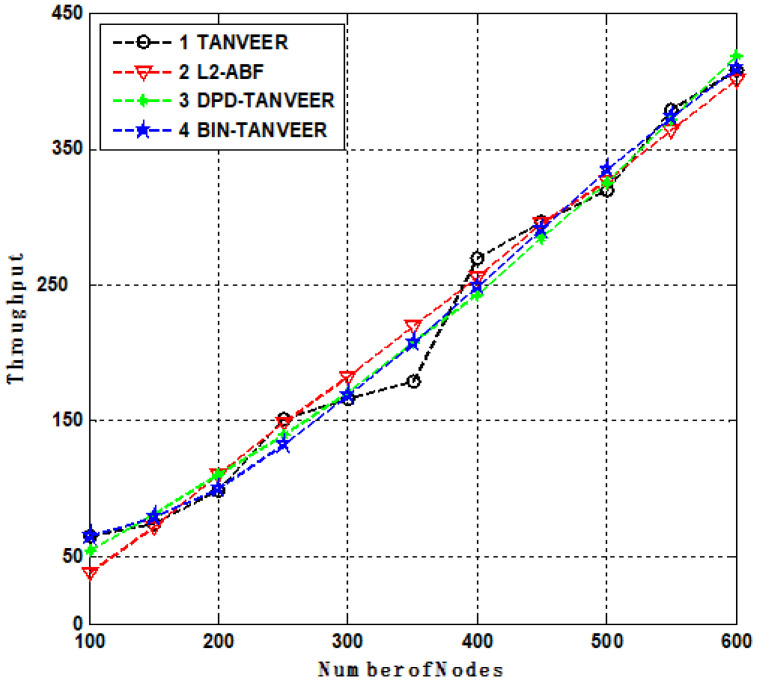
Throughput Analysis of TANVEER.

Figure 15 presents the throughput of the network that indicates the successful packets delivery from source to sonobuoys, and it is measured in bits/second (bps). The TANVEER have streamline throughput for 250 number of nodes with the throughput of 130 bps. As the number of nodes increased, the occurrence of empty regions starts, and the nodes are willing to finding the alternate path. In this situation, the TANVEER suddenly decreased up to 110 bps and then again increased up to 260 bps for the next 100 number of nodes, at this point, it was smooth throughout the network’s lifetime and achieved 370 bps for full number of nodes that are used under experiment. The DPD/BIN-TANVEER both depict the same behavior that started from 40 nodes with 45 bps and achieved 400 bps with 500 nodes, the reason is that as the empty region is found inside the network the routing path is changed and serve as BIN-TANVEER and the ratio of DPD is increased for the time being. In this case, the TANVEER consumes little more energy, but throughput is reached at 370 bps.

### 10.6. Influence of Coverage Area

**Figure 16:**
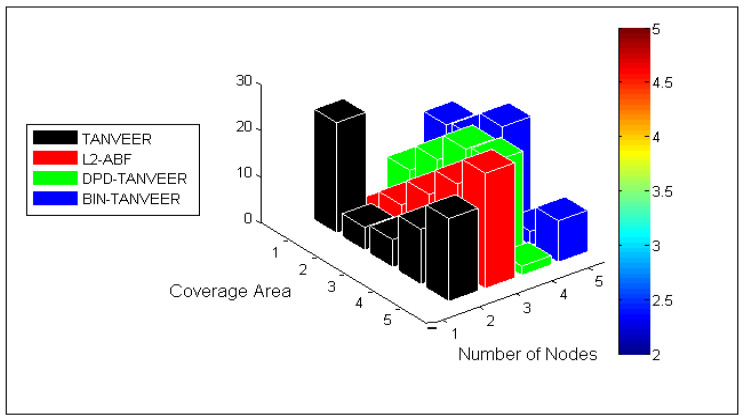
Analysis of coverage area.

From Figure 16, in order to verify the coverage area of the TANVEER, BIN-TANVEER, and DPD-TANVEER, we have conducted simulation against the number of nodes and coverage area. The slip of the area is also shown beside the simulation graph. From Figure 13, TANVEER has the highest coverage area among all other sub-sections, including the baseline approach. For example, the L2-ABF works with 500 nodes, and the quantity of nodes with TANVEER is 100 more nodes in order to cover the simulation environment and network volume.

L2-ABF used an angled approach while TANVEER works with tri-angular vector-based approach, and the DPD mechanism is more robust than the L2-ABF approach. Furthermore, the BIN technique that is novel in nature is only introduced with TANVEER, and the alternate path coped with binary internodes, hence the coverage area from source to sonobuoys was maximum as desired and that is depicted in Figure 16.

## 11. Comparative Performance Trade-Offs

The comparative performance analysis is presented in this section of our proposed scheme with a baseline approach. Our proposed scheme consists of the following: TANVEER, DPD-TANVEER, and BIN-TANVEER; furthermore, the LBA is another additional layout for deploying the number of nodes. The comparative approach L2-ABF is considered the angle-based flooding without knowing in advance either the next forwarder nodes is enough available for flooding or not, which is the reason every time the angle must be computed for forwarding the data.

Therefore, the overall consumption of energy is increased and there is minimum throughput. However, in the dense region, usually, the nodes not much consume energy near the anchored node, so the occurrence of empty region is not much observed in L2-ABF. On the other hand, TANVEER makes an angle for forwarding the data packets, and then it remains stable with the value of K and three possible directions. In the worst case, if the empty region is detected on one side of the network, the change mechanism shifted the delivery of data on the other side of the angle. In addition, we may know in advance about the availability of the next forwarding nodes that ready to take and send the data towards the sonobuoys. Also, it tries to select the nodes with minimum neighbor set in order to optimize the routing path with the cost of low-energy and end-to-end delay. In this case, with DPD-TANVEER achieved high PDR and throughput by avoiding the fraction of empty regions. The watchman nodes in BIN take data from both sides in case of empty nodes and adjust the transmission range towards the sonobuoys, but it increases the energy consumption. The related performance trade-offs are discussed in Table 3.

## 12. Conclusions and Future Work

In this paper, we have proposed TANVEER, with its three sub-sections LBA-TANVEER, DPD-TANVEER, and BIN-TANVEER, as a routing protocol to overcome the issues of minimum throughput and maximum energy consumption in the acoustic environment. The TANVEER improve energy consumption, PDR, and throughput through bypassing the empty regions, whereas LBA-TANVEER is used to assign the node ‘*ID*’ to accurate detection of empty regions. However, the use of LBA-TANVEER helps to control the topology of the network. On the other hand, DPD-TANVEER uses a path change mechanism with the help of the next availability of forwarder node and improved the data delivery ratio of the network. Moreover, it has a high throughput as compared to TANVEER. Finally, BIN-TANVEER uses watchman-based adjustment of finding the empty nodes and set the watchman-based transmission towards the sonobuoys. During the empty regions of DPD-TANVEER it fails to find out any forwarder and sends the data packet to the node that is going to die. In this case, BIN takes the data from the dying node and sends it to the nearest node in order to ensure that the next selected nodes are not further empty. All the results are performed in extensive simulation against the fraction of empty region, throughput, end-to-end delay, and packet delivery ratio. The output of simulation depicts that the proposed scheme performed well against the baseline approach in different parameters like energy, throughput, and PDR. Furthermore, the major parameter of avoidance of empty regions was also achieved as compared to L2-ABF.

In future work, we planned to handle the occurrence of empty regions using an artificial intelligence technique in which nodes itself memorize the remaining energy and are replaced with their nearest neighboring node. Furthermore, the transmission range and its adjustment of the nodes is done by using a heuristics technique, so in this way, it will improve parameters such as link quality, nodes status, and energy consumption.

## Figures and Tables

**Table 1:** Summary of possible Underwater Acoustic Sensor Network routing schemes as discussed in related work.

Routing Scheme/Protocols	Single/Multiple Copies	Empty Regions Types Considered	Features	Single/Multi Sink	Assumptions	Knowledge Required/Maintained	Achievements	Limitations
DFR [10]	Multiple	Bypass the empty regions while flooding	Allow to at least one node participation during detection of empty regions	Single sink	Free error-prone link quality by flooding	Whole network	Improved network architecture w.r.to Empty regions	Not every time bypass the empty region while flooding
ANA (CTC + DTC) [8]	Multiple	Centralized and distributed architecture is deploying for Empty regions	Long term propagation model has been used	Multi-sink	Depth-control mechanism is used	Whole network	90% PDR is achieved using DA + CTC and DA + DTC	Highly used of energy consumptions are found
GEDAR [9]	Single copy	Empty regions are found by fixing the depth adjustment	Greedy approach is used	Multi-sink	Geographic and opportunistic routing scheme is introduced	Own and Sink location	Empty regions are easily located. It calculates the new depth for the sonobuoys for the delivery of the data.	The selection of neighboring sets has some trade-offs
DCR [11]	Multiple	Similar to GEDAR but not fixing the depth	For topology control, vertical movements are introduced	Multi-sink	For depth adjustment, by checking the status of neighboring nodes	Own, 1-hop neighbors and sink info.	Throughput and lifetime are achieved; further long routes are also established by depth control adjustment.	For every time the depth is calculated for flooding, so large end-to-end delays occurred
SWARM-SEA [11]	Single copy	Similar to GEDAR and DCR with energy architecture SWARM	To know in advance about empty regions	Multi-sink	Hybrid approach by using the strategy of DCR and GEDAR	Own and Sink location info.	Improved the empty regions fixing as expectations	Thus, the scheme is run in hybrid mode, and complexities are increased in underwater nodes movements
VARP [12]	Multiple	4G mechanism including space and time used for avoidance of Empty regions	Used for monitoring purpose of empty regions using on-board pressure gauges	Single sink	Hop count, sequence number and dummy depth information is considered for finding the empty regions	Uses three parameters like hop count, sequence number and depth information for making the direct path to the closest sonobuoys	Works well for empty region, location and depth is directly control the onboard surface station.	Longest propagation delay occurs, at every hop count location does not be accurate for empty regions
L2-ABF [6,7]	Single copy	Using angle-based hierarchy the occurrence of empty regions neglectable	Every node calculated its flooding angle by angle cone towards the destination	Single sink	First, every node calculates the angle for next hop-count and then forwards the packets	Own cluster info. (1-hop)	No need to explicit location information about the nodes, IDs are assigned, and novel layer-by layer hierarchy is used	Before the flooding, each time angle zone is calculated, so the unexpected delays occur
AHH-VBF [13]	Single copy	Empty regions are detected by using hop-by-hop	Instead of angle, vector based forwarding approach is used	Single sink	Does not take any classical assumptions	Early radius information is required	Radios information is done by using acoustic wavelength, therefore no need to take extra calculation between source and destination	Consumption of battery issues are highly affected
H2-DARPPM [14]	Single copy	Hop-by-hop dynamic addressing is considered	Parametric values of the selection of nodes is based upon the reminding energy	n/a	For monitoring empty regions, the pipelines approach is used	Nodes location is required.	Easily identify any type of disturbance by pipeline approach	Approach is costly for network topology
VBVA [15]	Multiple	Radius information is considered for detection of empty regions for every hop-count	Vector based void avoidance is used to address the void routing table	Multi-sink	Vector shift and back pressure to tackle the void empty regions	Radios information of the nodes are required	It is the first void empty region protocol that works with 3-dimensional topology with mobile nodes.	While using back pressure to avoid the concave empty regions, this process is too long
EE-WBA [16]	Single copy	Energy efficient watchman-based monitoring is used to consider the empty regions	Novel nodes like watchman are deployed around the sink	Multi-sink	Watchman with its secondary nodes are easily coped with sink neighboring nodes	Deployment of the watchman is based upon nodes’ movement with multi-sink architecture	Easily identify the hotspot regions with watchman nodes and improve the PDR	The energy of watchman is counterpart throughout the network lifetime
NADEEM [17]	Multiple	Data are only forwarded to its nodes when the neighboring nodes is clear cut from void nodes	Two novel approaches like FA-NADEEM and TA-NADEEM are work for void nodes	Multi-sink	Data are only forwarded to its nodes when the neighboring nodes is clear cut from void nodes	Status of corresponding nodes on the forwarding route is required	Improved PDR, and detection of void nodes to some extent	Low network throughput, fails to find another routing path when the void nodes are present on a dedicated path
ABF [6]	Single copy	Angle-based flooding is used to tackle the empty regions	Acute Angle is calculating for flooding	Multi-sink	Every Angle calculation is based upon for flooding like 180/2	Angle cone	Improved end-to-end delay	Empty regions problem exists to some extent
DBR [18]	Single copy	Depth-based routing approach is used by calculating the depth before data flooding	Using depth easy to cope with shallow water	Multi-sink	Firstly, the proper depth is calculated by the available number of nodes	Depth area	Improved durability and stability of the network	Not every time the exact depth is calculated for flooding
EECOR [19]	-	Relay set determine the source nodes before sending/broadcasting	Relay source nodes scheme	-	Introducing relay nodes using fuzzy based relay section scheme for forwards the packets	-	Improve throughput and energy consumption ratio	High end-to-end delay
PERP [20]	-	Forwarding node selector is used	Trimming mechanism is adopted	-	Using a power efficient communication scheme	-	Improve the effectiveness and feasibility of power based routing	Propagation latency is high
Greedy routing [21]	Both single and multiple copy	Using location-based and location free routing for nodes position	Pre-determined location of nodes is achieved	-	Assume GPS-based information of nodes	Beacon-based and pressure-based protocols	Due to self-comparison, the realistic parameters are achieved	Finding information about nodes is too costly
Location Free Routing [22]	Both single and multiple copy	Location free link-state mechanism is used	Every node selects a one-hop neighbor within an area that guarantees progress toward a sink	-	Packet forwarding is performed hop-by-hop considering one or several routing metrics	Using link-state strategy used between nodes	Loop-free strategy also improves network topology	High data packet loss ratio, further link is breaking the network topology is disturbed
Co-EEORS [23]	Not information	Cooperative routing among nodes	Using the optimal relay section of nodes	Multi sink	Chose a relay and destination node	Relay node information is required	Superior for delivery of data packets	Throughput is minimum among compared technique EE-DBR
Hybrid routing [24,25]	Both single and multi-copy	Used opportunistic and geo-graphic routing protocols	Using optical acoustic hybrid OA-UWSN	-	Using SDMA, enable to take real-time video and image analysis	-	-	In this scheme, network topology changes frequently
Opportunistic Routing Protocol [26]	Both single and multi-copy	-	Number of neighbors participating for forwarding the data	-	Using Dempster–Shafer evidence theory-based opportunistic routing (EBOR) protocol	-	Improve network lifetime and packet delivery	High energy consumption
QL-EEBDG [27]	-	Q-learning based efficient data gathering scheme	Not information	-	Optimal next-hop forwarder	-	Improve energy parameters	High delay and PLR
DVRP [28]	Single copy	Diagonal and vertical path is used	Using two may communication	Single sink	Vertical path is easily supported with diagonal movement	Number of nodes on the diagonal path is required	Improved data delivery and throughput	Routing cache overhead
SMDBRP and AEDGRP [29]	Single copy	-	Using a mobile sink	Single sink	Dynamic topology for sink movement	-	Efficient data gathering mechanism	Trade-offs relationship exist
ASEDGRP [30]	Single copy	Atomic path shaped data route is proposed	Novel path adjustment is prepared	Multi sink	AUVs used for data gathering purposes	Localize information is required	Efficient path and optimize routing is achieved	Longest the path makes delay
3H-RM [31]	Multiple	Data are sent up to three hopes	By sending data to the destination, it first sends the data to the nearest nodes	Multi sink	Data packet delivery starts when the three hops are completed	Hope based nodes information required	Node reliability is achieved by using a hope-based mechanism	Not be energy efficient
EEEHR [32]	Multiple	Assume hole repelling scheme	Smaller cluster is formed to make a larger one	-	Equal sharing of load repelling	Using cluster-based node selection	Improve various parameters with LEACH	Trade-offs
DBR and L2-ABF [33]	Multiple	-	Both have used proper mechanism used for data flooding	Multi sink	Depth and angle-based information is required	-	Nagle and depth both are efficient for data delivery	Trade-offs relationship is existing
Watchman Based Flooding [34]	Single copy	Similar to ABF but angle is not calculated, watchman itself calculate where to flood for empty regions	Novel watchman-based flooding is proposed	Single sink	Multi sink architecture	Assume to be best routing flooding to enhance the ratio of un-empty regions	Successfully compared with state-of-the-art technique like L2-ABF, simulation results show the improvement in network lifetime.	Highly energy is needed to deploy this technique
Subnet Based Backup Assigning Algorithm (SBBA) [35]	Single copy	For avoiding the empty areas, the network is dividing into subnets	Subnet, subnet head and gateways are helping out in this regard	Single sink	-	Source to sink information	By subnetting easily handles the large traffic of the network and improve the minimum target of avoidance of void holes.	Formally verified the technique but the further need for simulation
Eneregy Aware Distributed Sink Algorithm (EADSA) [36]	Multiple	Distributed sink is deploying for this purpose	Energy of distributed sink is distributed to among all nodes	Single sink	Sink has dynamic nature form the routing EADSA	Only node location and its routing information is required	Successfully monitors as well as maintains the network architecture.	Empty regions are existing, but it does not be identified by EADSA
2H-ACK [37]	Multiple	For early realization, first 2 hops are required to take the decision	Hop-by-hop delivery is possible to receive the acknowledgments	Multi-sink	Acknowledge is counted from the nodes when the 2H are functional	Only ACK information is required	Find the nodes with minimal hops counts, only two.	Empty regions exist

**Table 2:** Simulation Evaluation Constraints.

Simulation Constraints	Values
Experimented Nodes	600
Network volume (m^3^)	2000 m × 2000 m × 2000 m
*T*_rang_ (m)	300
*P_tx_* (W)	2
Initial Energy (J)	100
*P_r_* (W)	0.2
Pi Idle power (W)	0.02
*F* (KHz)	10
*P_s_* (Bytes)	100
Baseline Approach	L2-ABF
Under Study Protocol	TANVEER

**Table 3:** Overall performance trade-offs.

Schemes	Features	Achieved Parameters	Trade-Offs
L2-ABF [6,7]	Geographic routing with layer-by-layer angle-based flooding for data communication underwater	Avoidance of empty regions results has increased the performance of the network	Maximum probability of void nodes and energy consumption for each iteration of angle computing
TANVEER	Geographic and opportunistic routing scheme using three-angle adjustment and watchman-based transmission	Increased PDR and throughput of the network by bypass the empty nodes/regions	Observed high end-to-end delay to maximum coverage area
LBA-TANVEER	Layer-based adjustment with data collision avoidance mechanism	Improved network topology and performance with adjustment of nodes	Not enough control, due to the dynamic nature of the environment but accurate detection of empty nodes is possible
DPD-TANVEER	Using path change mechanism by bypassing the empty regions	Improved PDR, throughput and fraction of empty regions	Due to path change, a long propagation delay occurred is not good
BIN-TANVEER	Works with novel watchman nodes using watchman-based transmission	Improved PDR and try to decrease PLR	Energy consumption is high

## Data Availability

Not applicable.
